# Characterization of Diabetic Retinopathy in Two Mouse Models and Response to a Single Injection of Anti-Vascular Endothelial Growth Factor

**DOI:** 10.3390/ijms24010324

**Published:** 2022-12-25

**Authors:** Tamar Azrad-Leibovich, Alon Zahavi, Moran Friedman Gohas, Myles Brookman, Orit Barinfeld, Orkun Muhsinoglu, Shalom Michowiz, Dror Fixler, Nitza Goldenberg-Cohen

**Affiliations:** 1 Krieger Eye Research Laboratory, Felsenstein Medical Research Center, Petach Tikva 4941492, Israel; 2 Sackler Faculty of Medicine, Tel Aviv University, Tel Aviv 6997801, Israel; 3 Department of Ophthalmology, Rabin Medical Center—Beilinson Hospital, Petach Tikva 4941492, Israel; 4 Laboratory of Eye Research, Felsenstein Medical Research Center, Petach Tikva 4941492, Israel; 5 Department of Neurosurgery, Rabin Medical Center—Beilinson Hospital, Petach Tikva 4941492, Israel; 6 Faculty of Engineering and Institute of Nanotechonology and Advanced Materials, Bar Ilan University, Ramat Gan 5200100, Israel; 7 Department of Ophthalmology, Bnai Zion Medical Center of Israel, Haifa 3339419, Israel; 8 Bruce and Ruth Rappaport Faculty of Medicine, Israel Institute of Technology—Technion, Haifa 3200003, Israel

**Keywords:** diabetic retinopathy, anti-vascular endothelial growth factor, neovascularization, transgenic mice

## Abstract

In this study, we characterized diabetic retinopathy in two mouse models and the response to anti-vascular endothelial growth factor (VEGF) injection. The study was conducted in 58 transgenic, non-obese diabetic (NOD) mice with spontaneous type 1 diabetes (n = 30, DMT1-NOD) or chemically induced (n = 28, streptozotocin, STZ-NOD) type 1 diabetes and 20 transgenic db/db mice with type 2 diabetes (DMT2-db/db); 30 NOD and 8 wild-type mice served as controls. Mice were examined at 21 days for vasculopathy, retinal thickness, and expression of genes involved in oxidative stress, angiogenesis, gliosis, and diabetes. The right eye was histologically examined one week after injection of bevacizumab, ranibizumab, saline, or no treatment. Flat mounts revealed microaneurysms and one apparent area of tufts of neovascularization in the diabetic retina. Immunostaining revealed activation of Müller glia and prominent Müller cells. Mean retinal thickness was greater in diabetic mice. RAGE increased and GFAP decreased in DMT1-NOD mice; GFAP and SOX-9 mildly increased in db/db mice. Anti-VEGF treatment led to reduced retinal thickness. Retinas showed vasculopathy and edema in DMT1-NOD and DMT2-db/db mice and activation of Müller glia in DMT1-NOD mice, with some response to anti-VEGF treatment. Given the similarity of diabetic retinopathy in mice and humans, comparisons of type 1 and type 2 diabetic mouse models may assist in the development of new treatment modalities.

## 1. Introduction

Diabetic retinopathy can lead to poor vision and even blindness due to many factors [1]. The suggested multifactorial underlying mechanism includes neuronal and vascular damage. The neurovascular complexes in the diabetic retina show neurochemical and electrical changes, leading to anatomical and functional impairment [2]. Vascular damage is caused by weakening of the retinal capillary wall by high glucose levels and consequent leakage of blood components into the surrounding space, leading to retinal thickening and relative ischemia, followed by proliferative growth of new vessels and macular edema [3].

Vascular endothelial growth factor (VEGF) is a major pathogenic factor in diabetic retinopathy and serves as an important therapeutic target [4]. Even patients with non-proliferative diabetic retinopathy are treated with intravitreal injections of anti-VEGF agents for macular edema [5]. The main anti-VEGF agents with well-established efficacy in this setting are bevacizumab (Avastin^®^), ranibizumab (Lucentis^®^), and aflibercept (Eyelea^®^) [5]. 

Several mouse models of diabetes have been investigated. Many researchers have chemically induced a state resembling insulin-dependent diabetes using peritoneal injections of streptozotocin (STZ), with varying protocols in terms of dosages and number of injections [6,7]. The most common transgenic mutant strain used is non-obese diabetic (NOD) mouse, which spontaneously acquires type 1 diabetes (DMT1) due to autoimmune destruction of insulin-producing pancreatic β-cells by CD4+ and CD8+ T cells [8]. Type 2 diabetes (DMT2) has been studied in db/db transgenic mice that are homozygous for a mutation in the leptin receptor gene. The lack of leptin, a satiety-signaling hormone, leads to obesity and hyperglycemia at age 4–8 weeks [9,10]. 

Diabetic mice are characterized by a loss of retinal microvessels, reduced retinal perfusion, and disordered focal vascular proliferation [11,12,13]. Only a few studies have investigated diabetic retinopathy in these models, and only a small minority of them has been focused on the development of neovascularization [9]. 

The aims of the present study were to characterize the clinical, histological, and molecular changes in the retina of NOD mice with DMT1 and transgenic db/db mice with DMT2 and to evaluate the response of the retinal edema to a single intravitreal injection of anti-VEGF medication. 

## 2. Results

**Figure 1:**
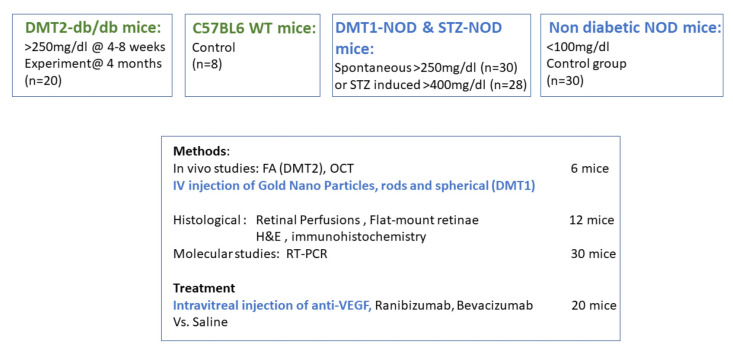
Experimental design of the study. Mice groups, study methods, and treatments are described in brief.

An experimental design depicting the main points of the study design is provided (Figure 1). 

### 2.1. Microaneurysms on Fundus Photography and Fluorescein Angiography (FA) 

**Figure 2:**
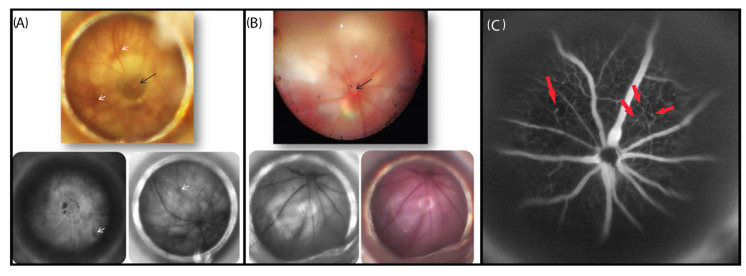
Color fundus photography and red-free and fluorescein angiography. (**A**) DMT1-NOD mice. (**B**) Control mice. The optic nerve head is indicated by a black arrow. White arrows indicate suspected leakages on angiography. The control non-diabetic eyes show normal retinal vasculature. Fluorescein angiography. (**C**) DMT2-db/db mice. Hyperfluorescent areas (red arrows) suggest focal vascular leakage.

Microaneurysms are the leading clinical marker of diabetic retinopathy. Tortuosity of the retinal vessels was noted on fundus photos and in fluorescein angiography (FA) imaging, with some leakage. However, there was no clear evidence of neovascularization on FA in DMT1-NOD mice (Figure 2A,B), whereas some leakage points were noted in DMT2-db/db mice (Figure 2C). 

### 2.2. Retinal Edema in Optical Coherence Tomography (OCT) and Histological Studies

Optical coherence tomography (OCT) showed thickening of the retina in DMT1 mice (320 microns) compared to non-diabetic mice (244 microns). Histological studies in untreated mice showed a mean retinal thickness at the midperiphery (500 microns from the optic nerve head) of 280 ± 43 µm in the DMT1-NOD diabetic groups, 299 ± 26 µm in the DMT2-db/db diabetic group, and 213 ± 50 µm in the control (non-diabetic) group. 

### 2.3. Tortuosity and Neovascularization in Retinal Perfusion and Histological Studies

**Figure 3:**
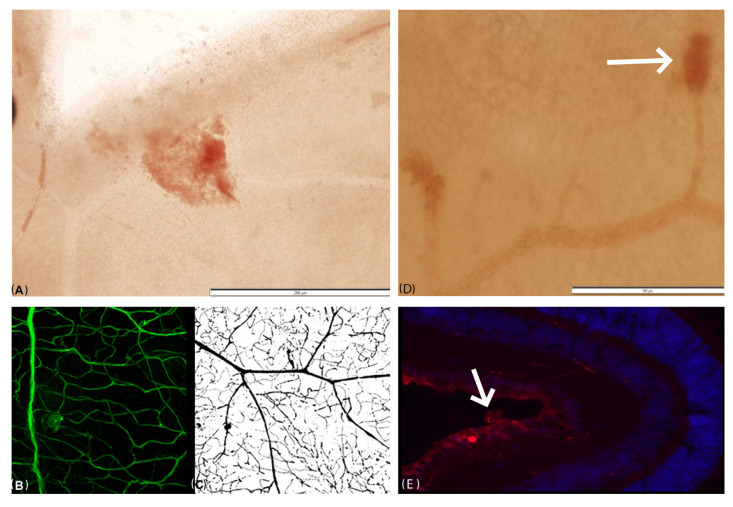
Perfusion studies with flat-mount retinas. (**A**) Flat-mount retina without perfusion. The intravitreal fan-shaped vascular bulging suggests pathological proliferation of retinal vessels. (**B**) Flat-mount retina perfused with fluorescence gel, demonstrating tortuosity of the vessels and an area suspected to be tufts of neovascularization. (**C**) Flat-mount retina perfused with India ink, showing sacculation of the retinal vessels. The findings suggest relaxation of the retinal wall and microaneurysms. Another DMT2 db/db mouse showed vascular leakage (**D**), with neovascularization noted upon sectioning (**E**).

Tortuosity of the retinal vessels was observed in retinal flat mounts of all diabetic mice; one appeared to have an area of tufts of neovascularization (Figure 3A,B). Microaneurysms were demonstrated with India ink (Figure 3C) and fluorescent gelatin. Another DMT2 db/db mouse showed vascular leakage (Figure 3D), and a third had neovascularization noted upon sectioning (Figure 3E). The retinal vasculature of the control mice was normal. 

### 2.4. Gold Nanoparticle (GNP) Detection/Nondetection with AirSEM and OCT Angiography (OCTA) 

**Figure 4:**
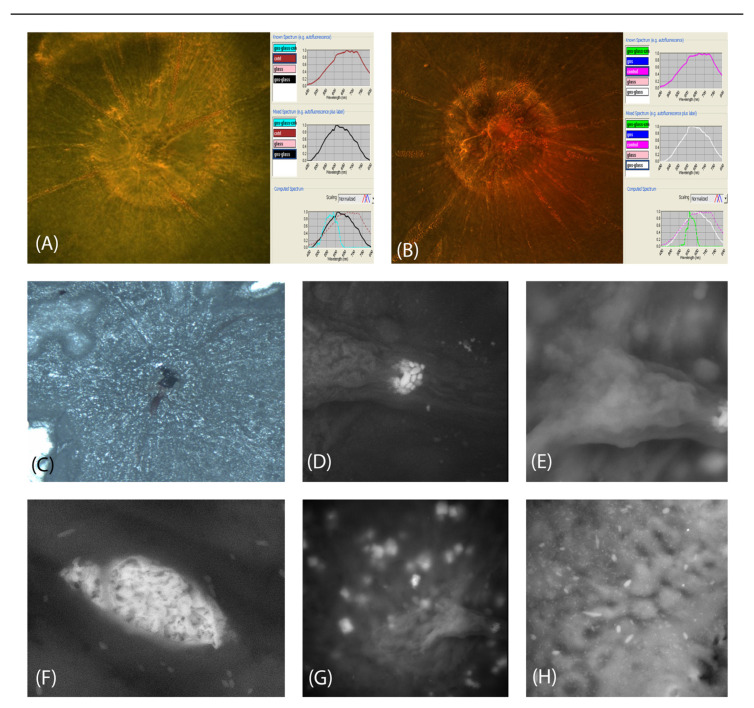
Hyperspectral microscopy. Non-obese, non-diabetic mice injected with intravenous GNPs. Hyperspectral dark-field ×200 imaging shows spherical GNPs (**A**) and rod-shaped GNPs (**B**) in the tissue but not in the vasculature. Air scan electron microscopy. DMT1 mouse following GNP injection. The low-magnification image (**C**) shows the entire retina with the optic nerve at the center. (**D**–**G**) Hyperfluorescence can be seen from cells and from GNPs in the tissue but not in the microvasculature (**H**).

Dark-field hyperspectral microscopy revealed gold nanoparticles (GNPs) in all evaluated mice. Both sphere- and rod-shaped GNPs were identified in the tissue but not specifically located in the microvasculature (Figure 4A,B). 

OCTA evaluation of GNP-injected mice failed to reveal blood flow or GNP flow, and AirSEM identified GNPs in the tissue but not specifically in the lumen of the retinal blood vessels (Figure 4C–H). 

### 2.5. GFAP and RAGE Expression upon Molecular Analysis

**Figure 5:**
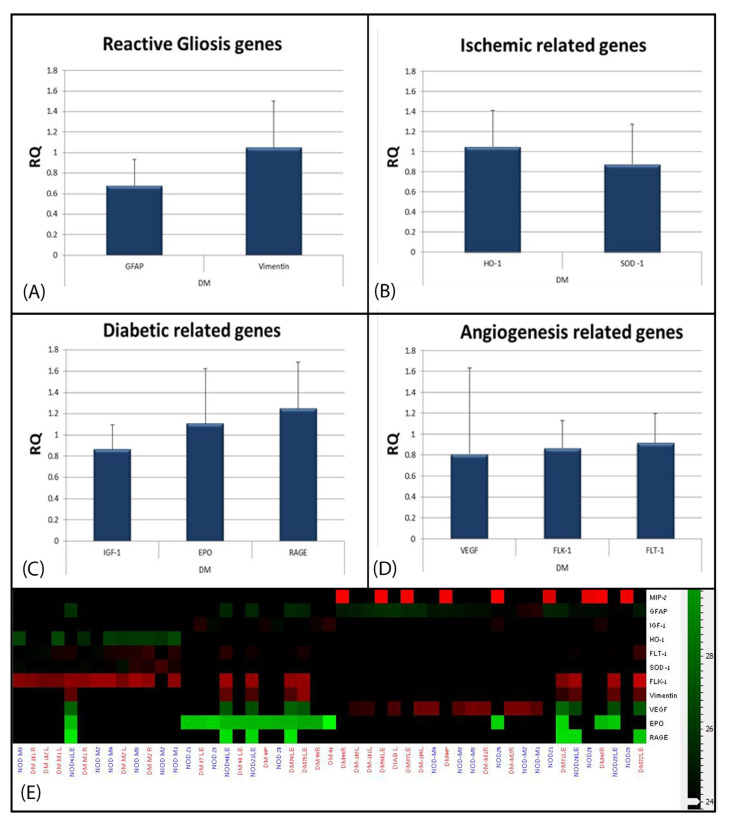
Molecular analysis of the diabetic retina in DMT1 mice. (**A**) Gliosis-related genes (GFAP and vimentin). GFAP levels were significantly reduced (0.35-fold, *p* = 0.04), and vimentin levels remained at baseline. (**B**) Oxidative stress-related genes (HO-1 and SOD-1). There was a statistically non-significant trend of a mild reduction in SOD-1 levels, with no change in HO-1. (**C**) Diabetes-related genes (EPO, IGF-1, and RAGE). EPO and IGF1 remained at baseline, and a statistically non-significant trend of a RAGE increase of 1.25-fold was noted. (**D**) Angiogenesis-related genes (VEGF-A, Flt-1, and Flk-1). No changes were noted. (**E**) Heat map of over- and underexpression of the genes investigated in this study. (ACTB served as the housekeeping gene).

The expression of oxidative stress-, angiogenesis-, gliosis-, and diabetes-related genes is shown in Figure 4A–E for all groups of mice. In contrast to the activation of Müller glia demonstrated by immunohistochemistry, upon molecular analysis, *GFAP* expression levels were significantly reduced in DMT1 mice (0.35-fold, *p* = 0.04) and a statistically non-significant trend of a mild increase in DMT2 diabetic mice of 1.75-fold was noted. Vimentin levels remained at baseline (Figure 5A). 

In the DMT1 mice, expression levels of *EPO* and *IGF-1* remained at baseline, whereas a statistically non-significant trend towards an increase in levels of RAGE was noted by 1.25-fold (Figure 4C). A statistically non-significant trend towards a mild reduction in *SOD1* levels was measured, with no change detected in the other oxidative stress or angiogenesis genes. 

### 2.6. Activation of Müller Glia with Prominent Müller Cells upon GFAP Staining

**Figure 6:**
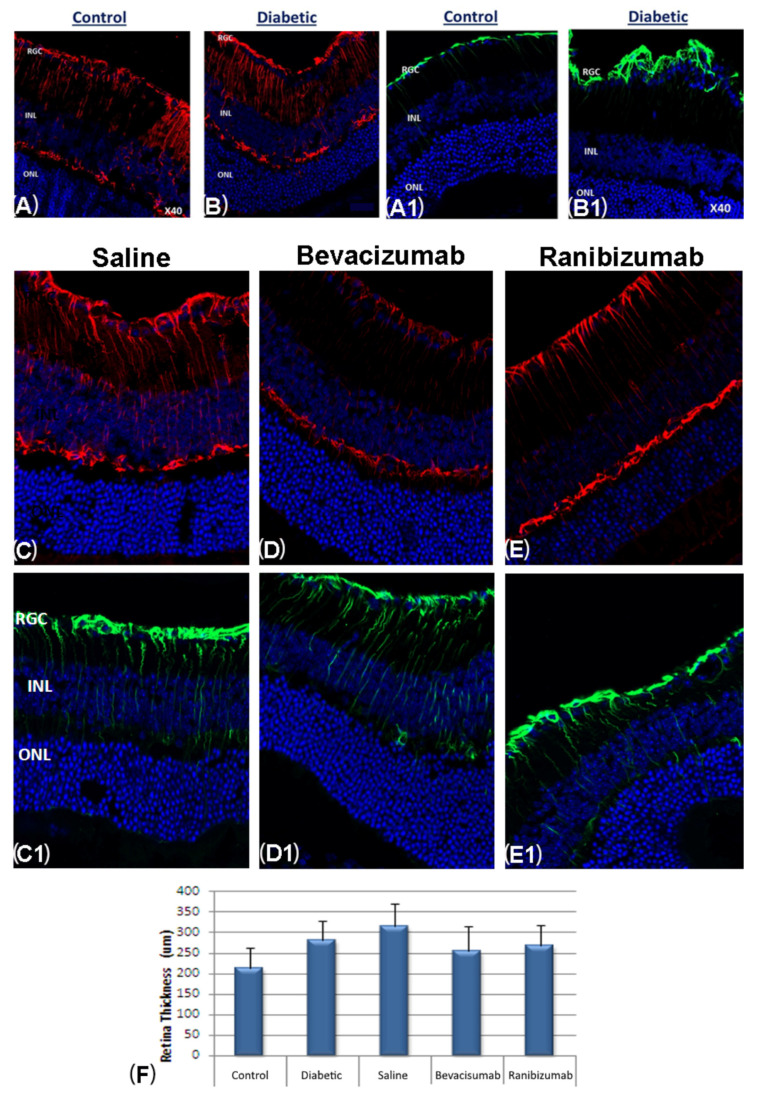
GFAP immunofluorescence staining and response to intravitreal injection of anti-VEGF. (**A**,**B**) Immunofluorescence staining with vimentin (red) in control mice (**A**) compared to DMT1-NOD mice (**B**). (**A1**,**B1**) Immunofluorescence staining for GFAP (green) in control mice (**A1**) compared to DMT1-NOD mice (**B1**). Together, these findings indicate the activation of Müller glia in the DMT1-NOD group. (**C**–**E**) Reduction in gliosis. Response of the control and DMT1-NOD groups, respectively, to a single injection of saline (**C**,**C1**), bevacizumab (**D**,**D1**), and ranibizumab (**E**,**E1**). (**F**) Retinal thickness in the control and untreated diabetic mice and in the mice treated with a single injection of bevacizumab, ranibizumab, or saline into the right eye. In the bevacizumab-treated mice, mean retinal thickness was 260 + 53 µm in the right eye and 241 ± 34 µm in the left eye; corresponding values for ranibizumab were 267.5 ± 47 µm and 223 ± 39 µm. Retinal thickness was 280 ± 43 µm in the untreated DMT1-NOD mice; 310 ± 53 µm in the saline-treated NOD mice and 273.6 ± 55 µm in the left eye; and 213 ± 50 µm in the non-diabetic NOD mice.

In contrast to the inverse GFAP expression between DMT1 and DMT2 diabetic mice, immunostaining for GFAP and vimentin showed activation of Müller glia and prominent Müller cells in the retina of all DMT1 mice (28 STZ-NOD) compared to the non-diabetic NOD mice (n = 7) (Figure 6A,B) but not in DMT2 mice. 

### 2.7. Reduced Gliosis and Retinal Edema following Intravitreal Injection of Anti-VEGF

The effectiveness of anti-VEGF treatment was evaluated in 28 DMT1-NOD mice divided into four equal groups. Five non-diabetic NOD mice served as controls. The 14 DMT1 mice treated with a single intravitreal injection of anti-VEGF agent (seven bevacizumab, seven ranibizumab) showed complete suppression of gliosis, whereas in the diabetic mice treated with a single injection of saline, gliosis remained at maximum (Figure 6C–E). The retinal thickness in non-diabetic control mice was 223.8 ± 29.7 µm, that NOD diabetic mice was 217.2 ± 37.2 µm (*p* = 0.00), and that diabetic db/db mice was 299.7 ± 26.7 µm (*p* = 0.00). Following bevacizumab injection in the right eye, retinal thickness measured 260 ± 53 µm in the right compared to 241 ± 34 µm in the left eye; following ranibizumab injection, retinal thickness was 267.5 ± 47 µm and 223 ± 39 µm; following saline injection, the values were 310 ± 53 µm and 273.6 ± 55 µm (Figure 6F). 

## 3. Discussion

In this study, we characterized the retinal vascular changes caused by DMT1 retinopathy in NOD mice and DMT2 retinopathy in db/db leptin-deficient mice. We found a high similarity to human diabetic retinopathy in terms of retinal vasculopathy, retinal edema, and thickening, as well as some response to anti-VEGF injection [9].

In previous studies of diabetes mouse models, although neovascularization was known to occur, its presence was not fully established, and rates were relatively low [12]. As approximately 20% of NOD mice acquire spontaneous hyperglycemia with high mortality without treatment, as shown in this study, few live long enough for proliferative diabetic retinopathy to develop. Given that the risk of development of neovascularization increases with time [12], partial control of glucose levels, as previously reported [12,14], may improve survival and enable improved characterization of this model [15]. In the present study, we did not detect neovascularization in FA, but using a large number of mice under close observation, we were able to do so in flat-mount retinas of db/db mice and in histological sections. Owing to the low rate of spontaneous diabetes in NOD mice, we also induced diabetes chemically in a subgroup of non-diabetic NOD mice. Although it was previously reported that STZ induction of DMT1 is not effective in young NOD mice, as it prevents and reverses islet destructive autoimmunity [7], we found that hyperglycemia (>250 mg/dL) developed within 5–7 days after STZ injection. DMT2 developed in all the db/db mice, as expected.

India ink and fluorescence gel demonstrated microvascular anatomical details better than FA. We found multiple microaneurysms using both methods. There were changes in the retinal vessels of the DMT1-NOD and db/db mice but not in the non-diabetic control mice. Similar findings were reported by Shaw et al. [16] and Li and Sun [17]. Fluorescence gel was associated with higher accuracy and fewer artifacts than India ink, making it possible for us to also identify tortuosity of the retinal vessels and tufts of focal proliferation. However, neovascularization was found in only one flat-mount retinal sample from a DMT1-NOD eye that was not perfused (Figure 2A). It was surprising to note neovascular tufts, which are a characteristic of late phases of diabetic retinopathy and are usually not observed in animal models, whereas extravasations were not observed.

GNPs are biocompatible and non-toxic, and their presence can be evaluated with imaging modalities including computed tomography and positron emission tomography [18]. Their use in ophthalmology has hardly been explored. We used spherical (20 nm) and rod-shaped (25 nm × 65 nm) GNPs. The absorption wavelength spectrum is 530 nm for spherical GNPs and depends on the orientation for rod-shaped GNPs. We hypothesized that GNPs would improve OCTA detection of red blood cell flow in the retinal vasculature, as reported by others using modified scanning parameters. When we applied the manufacturer’s parameters for human scans, we failed to visualize the retinal vasculature in mice both with and without injection of GNPs. However, we were able to detect the GNPs using flat-mount retinas and hyperspectral microscopy but could not locate GNPs in the vascular lumen or determine possible vasculopathy. This was also true under airSEM. Nevertheless, the detection of GNPs in the surrounding tissue implies possible future applications in ophthalmic research. Although others reported that intravenously administered GNPs were detectable in tissues under spectral microscopy [19], they used GNPs as tissue markers following fixation ex vivo or using techniques not applicable in ophthalmic research [20]. An in vivo attempt to use GNPs to demonstrate the retinal microvasculature has not been described before. 

Initially, hyperglycemia causes damage to endothelial cells in blood vessels of diabetic retinas, in addition to pericyte apoptosis [2]. Müller cells, an important participant in retinal angiogenesis, vascular leakage, and inflammatory reactions [2] undergo astrogliosis, as manifested by an increased content of intermediate filaments [2]. They respond to hyperglycemia by increasing the synthesis of glial fibrillary acid protein (GFAP) and vimentin and by producing inflammatory and angiogenic cytokines, such as hypoxia-inducible factor (HIF)-1. Accordingly, studies have reported an increase in HIF-1 in retinal inflammation, vascular leakage, and neovascularization [21]. HIF-1 also influences the level of expression of the oxidative stress-related *HO-1* and *SOD-1* genes [22]. Furthermore, chronic hyperglycemia increases oxidative stress and inflammation, which accelerate the formation and accumulation of advanced glycation end products (AGEs) and their receptor, RAGE [3]. This, in turn, induces Müller cells to upregulate the secretion of VEGF [4]. Studies have reported high levels of VEGF in the vitreous fluid of patients with proliferative diabetic retinopathy [4]. However, in rodent models of diabetes, vitreal VEGF levels fluctuate during the course of the disease [23]. 

Reactive gliosis in rodents is induced by Müller cells, mainly secondary to oxidative stress [24]. In the present study, the DMT1-NOD mice showed immunohistological activation of Müller glia by astrocyte- and Müller-cell-secreted GFAP but a significant reduction in GFAP mRNA levels upon molecular analysis. Gliosis was significantly reduced following anti-VEGF administration. In the DMT2-db/db mice, levels remained stable. In contrast, Masser et al. [25] reported an increase in GFAP both histologically and molecularly in a diabetic rat model, possibly due to genetic differences between the models and the timing of analysis following diabetes onset. Reactive gliosis is a dynamic process, with temporal changes mostly clustering into categories of immediate, delayed, and chronic. Vázquez-Chona et al. [26] found that histological and molecular GFAP results varied depending on the interval from the retinal injury. 

VEGF mRNA levels were not elevated in the retinal samples, despite a trend in histological evidence of reduced retinal thickness in response to a single anti-VEGF injection. This discrepancy may also be explained by the time at which each examination was performed, based on internal biofeedback, as (histologically detected) upregulation leads to reduced mRNA expression. In humans with diabetic retinopathy, vitreal VEGF levels have been shown to be increased [4]. 

Retinal thickening was observed in diabetic mice in our study, unlike in some previous studies [6,9]. Retinal edema in mice is difficult to accurately determine; therefore, the effect of anti-VEGF cannot be decisively concluded in relation to retinal edema differences. A longer duration of diabetes may result in more significant differences. However, treatment of the right eye with anti-VEGF seemed to affect the untreated eye compared to controls. This cross effect was stronger with ranibizumab than bevacizumab, in line with previous findings. Overall, both eyes of treated mice had thinner retinas than control mice (saline-treated and untreated). In the treated eyes, bevacizumab had a slightly better effect than ranibizumab. It was also better at alleviating gliosis relative to control (untreated) eyes, although the reason for this phenomenon is unclear. As ranibizumab is a smaller molecule with a shorter half-life, it is possible that its effect on the other eye is better. Additionally, there is a species-specific effect that might influence treatment response [11].

There was no difference between diabetic and non-diabetic NOD mice in the expression of other genes involved in hypoxia, inflammation, oxidative stress, and apoptosis that were evaluated, with the sole exception of the gene coding RAGE, a multiligand signal-transduction receptor of the immunoglobulin superfamily. *RAGE* was increased in the DMT1-NOD mice but not in the DMT2-db/db mice. Similar findings were reported in human and animal models [3,6,7,23]. RAGE activates nuclear factor κB, leading to the production of proinflammatory cytokines and reactive oxygen species in glial and microglial cells, and plays an important role in diabetic retinopathy [24].

The growing understanding of the importance of RAGE and its ligands in diabetic retinopathy has led to investigations of treatment with RAGE antagonists [22]. A preliminary study in a model of db/db transgenic mice [22] showed that the anti-RAGE-treated group had significantly fewer pericyte ghosts and better electrophysiological results than littermate controls. In other studies of mouse models with chemically induced DMT1, anti-RAGE treatment successfully blocked the development of capillary degeneration and the accumulation of albumin in the neural retina but did not affect the expression of intercellular adhesion molecule (ICAM)-1 [11]. The reason for the lack of change in RAGE levels in our db/db mouse model is not clear. 

This study is subject to several limitations. The reported effects are very mild, possibly due to the short periods during which mice were hyperglycemic, with high glucose values but also high mortality at DMT1, or the euthanization time following injection, which may have been prolonged [27]. Additionally, as previously described in the literature [12], NOD/ShiLtJ mice have an albino background lacking pigment and; therefore, FA cannot be performed adequately in this group (as the controls in this study were NOD mice without diabetes). Therefore, Indian ink in a flat mount was chosen to demonstrate retinal blood vessels in this group. Another limitation is that two different IHC staining methods for gliosis were carried out. However, these were insufficient to define subtle differences among the groups. Future studies may use Western blotting in addition to immunohistochemistry studies in order to quantify such differences.

## 4. Materials and Methods

### 4.1. Animal Model

This study was conducted in a university-affiliated medical center. All experiments were performed in accordance with the Association for Research in Vision and Ophthalmology (ARVO) Statement and were approved and monitored by the local Institutional Animal Research Committee. Both sexes were included in the study equally in each group. 

Eighty-eight transgenic NOD mice (NOD/ShiLtJ) aged 6 months were monitored for diabetes with weekly blood glucose level checks. Diabetes (glucose level >250 mg/dL) developed spontaneously in 30 mice (DMT1-NOD group) and was induced in 28 mice by an intraperitoneal (IP) injection of 100 mg/kg STZ (Sigma-Aldrich, Steinheim, Germany) after a 2 h fast under anesthesia with IP ketamine (80 mg/kg)/xylazine (4 mg/kg) (STZ-NOD group). The remaining 30 NOD mice without spontaneous or induced diabetes and 8 wild-type C57/bl6 (WT) mice served as controls. The average survival time from onset of diabetes was 2 months (range, 1–3.5 months). An additional 40 mice, all DMT1-NOD, died within 4 weeks or less from diabetes onset and were excluded. 

In addition, 20 transgenic DMT2-db/db homozygous mice (C57BLKS/J-lepr^db^/lepr^db^) aged 4 months (kindly provided by Professor Hochhauser, Laboratory of Cardiac Research, FMRC, Rabin Campus, Petach Tikva, Israel) were monitored for diabetes onset (glucose level >250 mg/dL). Repeated high-glucose measurements >250 mg/dL were recorded 2 months after diabetes onset. 

All 108 study animals underwent in vivo evaluations for vascular changes. Mice were euthanized with CO_2_ inhalation at age 5–8 months. The diabetic mice (DMT1-NOD, STZ-NOD, and DMT2 db/db) were euthanized after 14 days of uncontrolled high glucose levels, except those examined for response to intravitreal injection. Retinal perfusion studies were performed, and tissues were processed for histological studies, air-scanning electron microscopy, and molecular analysis. Anti-VEGF treatment was administered to DMT1-NOD mice at least 4 weeks after onset of uncontrolled hyperglycemia (>250 mg/dL), and the mice were euthanized 1 week later for histological and immunofluorescence evaluation. 

### 4.2. Clinical Evaluation of Retinal Vasculature

#### 4.2.1. Optical Coherence Tomography (OCT)

Anesthesia was induced with IP ketamine (80 mg/kg)/xylazine (4 mg/kg), followed by pupil dilatation with 0.5% tropicamide (Mydramid, Fisher Pharmaceutical Labs, Tel Aviv, Israel). OCT was performed with a spectral-domain system (Cirrus™ OCT, version 4.5.1.11 200 × 200 cube mode; Carl Zeiss Meditec, Dublin, CA, USA) using the human cornea protocol. 

#### 4.2.2. Fluorescein Angiography (FA)

FA was performed in 21 mice: 10 DMT1-NOD mice, 6 DMT2 db/db mice, 3 NOD controls, and 2 WT controls, as previously described by our group [23]. Prior to image acquisition, 0.04 mL of 25% sodium fluorescein (25% AMP; AK-Fluor, Akorn, Decatur, IL, USA) was injected IP. Color and red-free fundus photography and FA were performed immediately and up to 30 min later with a digital fundus camera (TRC 50× series; Topcon, Farmingdale, NY, USA) using a plastic contact lens customized for mice.

### 4.3. Retinal Perfusion Studies

Perfusion studies with fluorescent gelatin and India ink were performed in 5 diabetic DMT1-NOD mice and 5 controls. Gold nanoparticles were injected in 7 diabetic DMT1-NOD, 1 non-diabetic NOD, 4 DMT2 db/db, and 1 WT mice. 

#### 4.3.1. Fluorescent Gelatin

The method of fluorescent gelatin perfusion has been described previously [23,28,29]. In brief, mice were terminally anesthetized prior to infusion with IM ketamine/xylazine until the mouse no longer responded to painful stimuli. The mice were then infused with 5 mL of 2% gelatin and a mixture of heparin (1:5000; Bodene (Pty), Port Elizabeth, South Africa), atropine sulfate (1:10,000; Teva Pharmaceuticals, Petach Tikva, Israel), and fluorescein-conjugated bovine serum albumin (FITC-BSA). Thereafter, the mice were infused with 6 mL of 4% gelatin (Gelatin from porcine skin, #G1890; Sigma, St. Louis, MO, USA), together with the previous mixture. To coagulate the perfusions, the mice were placed in ice. The eyes were then enucleated and fixed immediately in 10% neutral buffered formalin in 0.1 M phosphate-buffered saline (PBS) at room temperature, and perfusion of the vasculature was examined. 

#### 4.3.2. India Ink

India ink (Higgins #4418; Sanford Corp, Bellwood, IL, USA) was injected into the left ventricle under deep terminal anesthesia. This method is well-established for retinal vasculature tracing, as described previously [30].

#### 4.3.3. Gold Nanoparticles (GNPs)

GNPs were designed and produced for the study at the Faculty of Engineering and the Institute of Nanotechnology and Advanced Materials, Bar Ilan University, Israel. Mice were placed under anesthesia as detailed above, and sphere- and rod-shaped GNPs (100 µL, 20 µgr/mL) were administered intravenously. OCT angiography (OCTA) (Optovue Inc., Fremont, CA, USA) was used to detect GNPs in the retinal circulation in vivo.

### 4.4. Retinal Flat-Mount Studies

#### 4.4.1. Fluorescence Microscope

As previously described, retinas was prepared as a flattened whole on a glass slide with a coverslip [31]. Images were obtained with a fluorescence microscope using appropriate filters for FITC-BSA (ApoTome, Carl Zeiss Microscopy, Jena, Germany).

#### 4.4.2. AirSEM

Flat-mount retinas of 4 DMT1-NOD diabetic GNP-injected mice were prepared as above without a coverslip and analyzed under airSEM^TM^*,* an innovative high-resolution scanning electron microscope (courtesy of B-Nano, Rehovot, Israel).

#### 4.4.3. Hyperspectral Imaging

Samples from GNP-injected mice (1 NOD non-diabetic, 3 DMT1-NOD diabetic, 1 WT, and 1 DMT2-db/db mice) were analyzed with a Cri Nuance^TM^ Hyperspectral Imaging System (Perkin Elmer, Hopkinton, MI) using a halogen light source (UN2-PSE100, Nikon, Tokyo, Japan) and a 40× objective (0.75 NA) and a 32-bit ultrasensitive charge-coupled device camera detector (N-MSI-EX) for imaging in the color code chart red–green–blue (RGB) mode. 

### 4.5. Histological Studies

#### 4.5.1. Hematoxylin and Eosin

As previously described, 10 µm thick sagittal sections were stained with hematoxylin and eosin and examined under a light microscope [31]. Total retinal thickness was measured in various fields of each section and in 3 sections of every 10 slides (30 consecutive sections), for a total of 7–10 slides per eye. 

#### 4.5.2. Immunostaining

Slides were washed in PBSX1 and PBS-TWEEN 20 0.5% (Sigma-Aldrich, St. Louis, MO, USA). After incubation in BSA, slides were incubated overnight at 4 °C with the following primary antibodies: anti-GFAP (1:200,04-1062, rabbit anti-mouse; Merck Millipore, Merck KgaA, Darmstadt, Germany) or anti-vimentin (1:500, AB-5733, chicken anti-mouse; Merck Millipore). Sections were washed and incubated for 1 h at room temperature with the following secondary antibodies: goat anti-rabbit IgG Alexa Fluor 488 (1:200, A11008, Molecular Probes, Invitrogen, Carlsbad, CA, USA) and goat anti-chicken IgG NL-557 (1:200,NL016; R&D Systems-Biotest, Minneapolis, MN, USA). They were then stained with NucBlue (#R37605; Molecular Probes, Eugene, OR, USA) and covered with ProLong Gold Antifade Mountant (#P36930; Molecular Probes). Slides were shielded from light and maintained at 4 °C. Images were obtained using a fluorescence microscope equipped with a DAPI filter (ApoTome; Carl Zeiss Microscopy, Jena, Germany).

### 4.6. Molecular Analysis

Retinas of 15 DMT1-NOD diabetic mice, 9 DMT2-db/db mice, and 15 non-diabetic mice were analyzed for genes involved in oxidative stress (superoxide dismutase 1 (*SOD1)* and heme oxygenase 1 *(HO1*)), angiogenesis (vascular endothelial growth factor A (*VEGF-A*)*,* vascular endothelial growth factor receptor 1 (*VEGFR-1)(Flt-1*), and vascular endothelial growth factor receptor 1(*VEGFR-2) (Flk1*)), gliosis (glial fibrillary acidic protein (*GFAP)* and vimentin (*VIM*)), and diabetes (receptor for advanced glycation end products (*RAGE),* insulin-like growth factor-I (*IGF-1*)*,* and erythropoietin (*EPO*)). 

#### Real-Time Quantitative PCR (RT-qPCR)

Retinas were separated under a microscope, incubated on RNAlater (Invitrogen), and maintained at −80 °C. Total RNA was extracted using TRIzolTM reagent (Invitrogen) according to the manufacturer’s protocol and then reverse-transcribed into cDNA using a-capacity cDNA RT kit +RNAse inhibitor (#4374967; Applied Biosystems, Foster City, CA, USA) according to the manufacturer’s instructions.

Following cDNA synthesis, retinal expression of mRNA of the 10 genes of interest was evaluated by RT-qPCR using gene-specific primers (Table 1). Reaction efficiency was tested against a standard curve for the retina. Gene expression was normalized to mouse beta actin, a housekeeping gene. Fast SYBR^®^ Green Master Mix (4385612 Applied Biosystems, Foster City, CA) and StepOneTM Software v2.2.2 (Applied Biosystems) were used. Reactions were performed in a 10 µL volume containing 1 µL cDNA, 10 µM/0.5 µL of each of the forward and reverse primers, and buffer included in the Master Mix. 

Cycling conditions were initial denaturation at 95 °C for 20 s followed by 40 cycles of 30 s at 95 °C and 30 s of annealing and extension at 60 °C. Duplicate RT-qPCR reactions were performed for each gene to minimize individual tube variability, and an average was taken for each time point. Threshold cycle efficiency corrections were calculated, and melting curves were obtained using cDNA for each individual-gene PCR assay. The results were quantified by the comparative threshold cycle (Ct) method (2-ΔΔCt method, where ΔΔCt = ΔCt (sample)—ΔCt (reference gene) (DATA assist TM Software v2.2.2, Applied Biosystems)). 

### 4.7. Intravitreal Injection of Anti-VEGF

Hyperglycemic DMT1-NOD mice were placed under anesthesia with IP ketamine (80 mg/kg)/xylazine (4 mg/kg) and topical oxybuprocaine hydrochloride 0.4%. Bevacizumab (75 µg/3 µL), ranibizumab (30 µg/3 µL), or saline 0.9% (3 µL) was injected in the right eye using a Hamilton syringe. The left eye served as an internal control. 

### 4.8. Statistical Analysis

Experimental data are expressed as mean and standard error. Student’s *t*-test was used to determine the significance of differences between groups. A *p* value < 0.05 was considered significant.

### 4.9. Data and Resource Availability

The datasets generated during and/or analyzed during the current study are available from the corresponding author upon reasonable request. No applicable resources were generated or analyzed during the current study.

## 5. Conclusions

In summary, the retinas of diabetic mice are characterized by vasculopathy and edema. Although there was no increase in retinal levels of *VEGF* or *GFAP* mRNA upon molecular analysis, in the NOD mice with DMT1 (spontaneous or STZ-induced), immunofluorescence studies revealed activation of Müller glia, and there was a good response to anti-VEGF treatment. Bevacizumab was slightly more effective than ranibizumab. In the DMT2-db/db mice, there was no activation of Müller glia. The lack of neovascularization upon FA in our model may be due to the short life span of the study animals after the development of diabetes. *GFAP* was the only diabetes gene tested that was significantly decreased in the DMT1-NOD mice and mildly increased in the DMT2 db/db mice. *RAGE* was increased but in the DMT1-NOD mice but not significantly. The ability to compare histological and molecular findings and to track new molecular pathways may lead to improvements in treatment modalities.

## Figures and Tables

**Table 1:** List of primers used for RT-PCR analysis.

	Gene	F	R
Angiogenesis	VEGF-A	CACGACAGAAGGAGAGCAGAA	CGCTGGTAGACATCCATGA
	Flt-1 (VEGFR1)	CCCCTCCCCAGAAATCGT	CAAATAGCGAGCAGACTTCAATG
	Flk-1 (VEGFR2)	CGCGGCCAAGAGGTTT	GATGCCAGCAAGTGGGAATT
Ischemia	HO-1	CAGGTGTCCAGAGAAGGCTTT	TCTTCCAGGGCCGTGTAGAT
	SOD-1	GCCCGGCGGATGAAGA	CGTCCTTTCCAGCAGTCACA
Activation of Müller Glia	GFAP	CGGAGACGCATCACCTCTG	TGGAGGAGTCATTCGAGACAA
	Vimentin	CAGCAGTATGAAAGCGTGG	GGAAGAAAAGGTTGGCAGAG
Diabetes-Related	IGF-1	AGAGACCCTTTGCGGGGC	CGGATAGAGCGGGTGCTT
	EPO	GCCCTGCTAGCCAATTCC	GGCGACATCAATTCCTTCTG
	RAGE	CTTGCTCTATGGGGAGCTGTA	GGAGGATTTGAGCCACGCT

## Data Availability

The data sets generated during and/or analyzed during the current study are available from the corresponding author upon reasonable request. No applicable resources were generated or analyzed during the current study.
